# Sex differences in QTc prolongation and electrocardiographic changes before and after antipsychotic treatment of schizophrenia

**DOI:** 10.3389/fpsyt.2026.1988458

**Published:** 2026-10-20

**Authors:** Xinwang Song, Fangcheng Fan, Jiaquan Liang

**Affiliations:** 1 Department of Psychiatry, Lianzhou Ningkang Hospital, Qingyuan, Guangdong, China; 2 Department of Psychiatry, The Third People’s Hospital of Foshan, Foshan, Guangdong, China

**Keywords:** electrocardiogram, QTc interval, QTc prolongation, schizophrenia, sex differences

## Abstract

**Introduction:**

Sex differences in ventricular repolarization are well established, with women showing longer corrected QT (QTc) intervals and greater susceptibility to drug-induced QT prolongation, but longitudinal data on sex differences in electrocardiographic (ECG) changes during antipsychotic treatment in acutely hospitalized patients with schizophrenia (SCZ) are scarce.

**Methods:**

We included 586 inpatients with SCZ (314 males, 272 females) who underwent ECG and metabolic assessments at admission and before discharge. ECG parameters, lipids and fasting glucose were compared between sexes, and QTc prolongation was defined using sex-specific thresholds (males >440/450 ms, females >460/470 ms). Analyses included paired and independent t-tests, McNemar tests, ANCOVA and multivariable logistic and linear regression, together with pre-specified sensitivity analyses using four alternative rate-correction formulae (Fridericia, Framingham, Hodges and Rautaharju), mixed-effects models and Benjamini-Hochberg correction; 95% confidence intervals (CIs) were reported for proportions.

**Results:**

At baseline, females had longer QTc than males (430.3 ± 23.3 vs. 416.6 ± 23.9 ms; P < 0.001), a difference consistent across all five formulae, whereas males had wider QRS duration (91.6 ± 10.2 vs. 86.0 ± 9.2 ms; P < 0.001). Marked QTc prolongation at admission was more frequent in males (5.4% [95% CI 3.4–8.5] vs. 1.5% [0.6–3.7]; Fisher exact P = 0.013). After treatment, QTc shortened significantly in males (−4.6 ms; P = 0.0004) but not in females (−2.2 ms; P = 0.165); this crude between-sex difference was not significant and its magnitude was formula-dependent, whereas the baseline-adjusted between-sex difference in ΔQTc remained significant across all formulae (female β = +6.5 to +9.8 ms; all P < 0.001). Male marked prolongation fell from 5.4% to 0.6% (exact McNemar P = 0.0007), and female sex was independently associated with lower odds of QTc prolongation tendency (odds ratio [OR] = 0.49, 95% CI 0.29–0.85).

**Discussion:**

In hospitalized patients with SCZ, females had longer baseline QTc and, in adjusted models, less QTc shortening, whereas males showed a higher prevalence of threshold-defined marked prolongation at admission. Because these exploratory findings are partly dependent on the rate-correction formula, sex-specific ECG monitoring should be evaluated prospectively.

## 1 Introduction

Schizophrenia (SCZ) is associated with a substantially elevated risk of premature mortality, with cardiovascular disease representing a leading contributor (1, 2). Patients with SCZ have overall increased risk of cardiovascular mortality compared with the general population, driven by both lifestyle factors and the adverse metabolic and cardiovascular effects of antipsychotic medications (3). Among drug-related cardiovascular risks, corrected QT (QTc) interval prolongation is of particular concern because it predisposes to torsades de pointes (TdP) and sudden cardiac death (4–6). Antipsychotics differ in their propensity to prolong the QTc interval: clozapine and olanzapine were associated with a higher incidence of QTc prolongation in a recent real-world inpatient study, and antipsychotics are among the drug classes most frequently implicated in QT prolongation in pharmacovigilance analyses (7, 8).

Sex is a fundamental determinant of ventricular repolarization. Population-based and large normative ECG cohort studies have consistently demonstrated that women have longer QTc intervals than men from adolescence to menopause (9–11), a difference attributable to the QT-shortening effect of testosterone and the modulatory effects of estrogens on cardiac potassium channels (9, 10). Women also account for approximately two-thirds of drug-induced TdP cases, and female sex has long been regarded as an independent risk factor for drug-induced long QT syndrome (12–14). In patients with SCZ receiving antipsychotics, several cross-sectional studies have reported that female sex is associated with a higher prevalence of QTc prolongation (15–17).

However, several gaps remain. First, many previous studies were cross-sectional, assessing QTc at a single time point during long-term treatment (18); longitudinal data on sex differences in electrocardiographic (ECG) changes from pre-treatment baseline through acute hospitalization are limited. Second, although QTc prolongation thresholds are sex-specific in clinical guidelines (males >450 ms, females >470 ms) (4), numerous studies applied a uniform threshold, which may obscure sex-specific risk patterns. Third, the interaction between sex and drug-specific QTc effects in Chinese hospitalized patients has rarely been examined. Sex differences in antipsychotic pharmacokinetics and adverse effects—including higher blood concentrations and greater metabolic burden in women—further support the need for sex-stratified cardiovascular safety analyses (19, 20).

Given these considerations, we conducted this study in 586 hospitalized patients with SCZ who underwent ECG and metabolic assessments both at admission and before discharge. We aimed to (1) characterize sex differences in baseline ECG parameters (2); compare changes in ECG and metabolic parameters during antipsychotic treatment between sexes (3); evaluate the prevalence and sex-specific patterns of QTc prolongation; and (4) identify factors associated with QTc prolongation and QTc changes. Given the observational design and the planned number of comparisons, the study was framed as exploratory.

## 2 Materials and methods

### 2.1 Study design and participants

This study included patients with SCZ who were hospitalized in two psychiatric hospitals—Lianzhou Ningkang Hospital and the Third People’s Hospital of Foshan—between January 2023 and December 2025 and had both admission and discharge ECG records. Inclusion criteria were (1): a clinical diagnosis of SCZ according to Diagnostic and Statistical Manual of Mental Disorders, Fifth Edition (DSM-5) (2); age 18–60 years (3); complete ECG and metabolic assessments at both admission (pre-treatment) and before discharge (post-treatment). Exclusion criteria were (1): known organic heart disease or congenital long QT syndrome (2); use of other QT-affecting medications (class III antiarrhythmics, macrolides) documented at admission (3); incomplete essential data. A total of 586 patients (314 males, 272 females) were included. The study was approved by the Ethics Committee of The Third People’s Hospital of Foshan (approval number: FSSY-LS202326) and all participants provided written informed consent.

### 2.2 Data collection

Demographic and clinical data were extracted from medical records, including age, sex, length of hospital stay, education level, and antipsychotic medication(s) at admission. The following ECG parameters were recorded at admission and before discharge: heart rate (beats per minute, bpm), QRS duration (ms), PR interval (ms), and QTc interval (ms). Metabolic parameters included triglycerides (TG), total cholesterol (CHOL), high-density lipoprotein cholesterol (HDL), low-density lipoprotein cholesterol (LDL) and fasting blood glucose (FBG).

### 2.3 ECG acquisition and measurement

Standard 12-lead resting ECGs were recorded with the patient in the supine position after at least 5 minutes of quiet rest, using the same model of electrocardiograph and an identical acquisition protocol at both participating hospitals. All tracings were analyzed with the built-in automated measurement algorithm of the electrocardiograph, which reports heart rate, PR interval, QRS duration and QT interval; the QT interval was measured from the onset of the QRS complex to the end of the T wave, preferentially in leads II and V5, and QTc was derived using Bazett’s formula (QTc = QT/√RR). Automated measurements may be biased when the T-wave end is ill-defined or when the signal is noisy (21), and the reliability of R-T measurement depends on the delineation algorithm (21), and different automated algorithms can yield systematically different estimates of QT and its variability (22). Therefore, all recordings were visually inspected, any tracing with excessive noise, baseline artefact or an ill-defined T wave was repeated, and values flagged by the algorithm were over-read by a clinician. The same measurement approach was applied at admission and before discharge in every patient. One admission or discharge ECG record contained physiologically implausible values and was treated as missing, so that analyzable ECG data were available for 585 patients (313 males, 272 females). ECGs were not available in digital form for the recalculation of QT from the raw waveform; consequently, the sensitivity analyses described below were performed on the automatically reported QTc by internally back-calculating the uncorrected QT interval (QT = QTc (Bazett) × √RR) and then re-applying each alternative correction formula.

### 2.4 Heart-rate correction

The QTc interval was automatically calculated using the Bazett formula (QTc = QT/√RR), the most widely used rate-correction method in clinical practice (23). Although guideline consensus recommends linear regression-based or alternative rate correction over Bazett’s formula because of its residual heart-rate dependence (23, 24), Bazett-corrected QTc values remain directly comparable with those reported in most previous psychiatric studies (15, 16). Because no single correction formula is universally accepted (24, 25), and because Bazett’s residual rate dependence could generate an apparent sex difference in QTc change when the two sexes differ in their heart-rate response, we additionally recomputed QTc using the Fridericia, Framingham, Hodges and Rautaharju formulae in pre-specified sensitivity analyses.

Antipsychotic medications at admission were classified into monotherapy groups (clozapine, olanzapine, risperidone, aripiprazole, amisulpride, quetiapine and others) and combination therapy.

### 2.5 Definitions

QTc prolongation was defined using sex-specific thresholds recommended for drug safety monitoring: QTc > 450 ms in males and > 470 ms in females for marked prolongation; and QTc > 440 ms in males and > 460 ms in females for prolongation tendency (4, 23). ΔQTc was defined as post-treatment QTc minus pre-treatment QTc.

### 2.6 Statistical analysis

All statistical analyses were performed using SPSS 26.0 and Python 3.12 (statsmodels and SciPy libraries). Continuous variables are presented as mean ± standard deviation (SD) and were compared between sexes using independent samples t-tests. Categorical variables are presented as frequencies (percentages) with 95% confidence intervals (CIs, Wilson method) and were compared using chi-square tests and Fisher’s exact test. Within-group changes from admission to discharge were assessed using paired t-tests, and sex differences in the magnitude of change (Δ) were tested using independent t-tests and, to model the two-factor (sex × time) repeated-measures design explicitly, mixed-effects models with a random intercept for participant were fitted for each parameter and the sex × time interaction was tested. To account for potential confounders, analysis of covariance (ANCOVA) was performed on the change in each parameter (post-treatment minus pre-treatment), with sex as the main factor and age, length of hospital stay and baseline value as covariates. Multivariable logistic regression identified factors associated with QTc prolongation tendency (male >440/female >460 ms), with sex, age, length of stay and antipsychotic drug group entered as covariates. Multivariable linear regression identified factors associated with ΔQTc, including sex, age, length of stay, baseline QTc and drug group. Drug group was entered as a categorical variable with risperidone monotherapy as the reference; patients receiving other single antipsychotics (aripiprazole, amisulpride, quetiapine or others) were collapsed into an ‘other drugs’ category, and combination therapy formed a separate category.

### 2.7 Sensitivity and robustness analyses

To address the rate-dependence of Bazett’s formula, ΔQTc and its between-sex difference were recomputed using four alternative correction formulae (Fridericia, Framingham, Hodges and Rautaharju), and the baseline-adjusted between-sex difference was re-estimated for each formula; within-sex paired changes and threshold-based prevalences were also examined for each formula. Because nearly half of the cohort received combination therapy, and because individual antipsychotics differ in their QTc liability, combination therapy was re-classified according to whether the regimen contained a higher-QTc-risk agent (clozapine or olanzapine), as defined by the literature (7, 8), and the regression models were repeated using this binary exposure in place of the drug group. Finally, to limit the risk of false-positive findings across the family of between-sex comparisons, the Benjamini-Hochberg false-discovery-rate (FDR) procedure was applied within each family of tests (baseline comparisons and change comparisons separately). Given the observational design, the exploratory nature of the analyses, and the sensitivity of threshold-based measures to the correction formula, the two pre-specified primary endpoints were (i) the between-sex difference in baseline QTc and (ii) the between-sex difference in ΔQTc; all other comparisons are reported as exploratory.

## 3 Results

### 3.1 Baseline characteristics

**Table 1** summarizes the baseline demographic and clinical characteristics of the study population by sex. The cohort comprised 314 males (53.6%) and 272 females (46.4%), with a mean age of 43.0 ± 11.5 years. Males were significantly younger (41.6 ± 11.7 vs. 44.6 ± 11.0 years, P = 0.002) and had longer hospital stays (90.2 ± 46.7 vs. 78.0 ± 53.2 days, P = 0.003) than females. Education level distribution did not differ significantly between sexes (P = 0.072), nor did the distribution of antipsychotic medications (P = 0.075).

**Table 1:** Baseline demographic and clinical characteristics of patients with schizophrenia by sex.

Variable	Male (n = 314)	Female (n = 272)	Test statistic	P value
Age (years)	41.6 ± 11.7	44.6 ± 11.0	−3.14	0.002
Length of hospital stay (days)	90.2 ± 46.7	78.0 ± 53.2	2.96	0.003
Illiterate/primary school or below, n (%)	50 (15.9%)	55 (20.2%)		
Junior high school, n (%)	122 (38.9%)	100 (36.8%)		
Senior high/technical secondary, n (%)	95 (30.3%)	62 (22.8%)		
College or above, n (%)	46 (14.6%)	54 (19.9%)	7.01	0.072
Clozapine, n (%)	32 (10.2%)	49 (18.0%)		
Olanzapine, n (%)	41 (13.1%)	32 (11.8%)		
Risperidone, n (%)	57 (18.2%)	51 (18.8%)		
Aripiprazole, n (%)	9 (2.9%)	15 (5.5%)		
Amisulpride, n (%)	8 (2.5%)	6 (2.2%)		
Quetiapine, n (%)	6 (1.9%)	7 (2.6%)		
Other antipsychotics, n (%)	9 (2.9%)	4 (1.5%)		
Combination therapy, n (%)	147 (46.8%)	106 (39.0%)	12.87	0.075
No record, n (%)	5 (1.6%)	2 (0.7%)		
Heart rate (bpm)	83.9 ± 17.0	85.8 ± 16.4	−1.36	0.176
QRS duration (ms)	91.6 ± 10.2	86.0 ± 9.2	6.83	<0.001
PR interval (ms)	150.1 ± 20.5	144.8 ± 18.5	3.25	0.001
QTc interval (ms)	416.6 ± 23.9	430.3 ± 23.3	−7.02	<0.001
Triglycerides (mmol/L)	1.6 ± 1.3	1.5 ± 1.3	1.65	0.100
Total cholesterol (mmol/L)	4.3 ± 1.1	4.8 ± 1.0	−4.93	<0.001
HDL cholesterol (mmol/L)	1.2 ± 0.3	1.5 ± 0.5	−8.66	<0.001
LDL cholesterol (mmol/L)	2.8 ± 0.9	3.0 ± 0.8	−2.05	0.041
Fasting blood glucose (mmol/L)	5.6 ± 1.4	5.8 ± 1.4	−2.18	0.030

Data are presented as mean ± SD or n (%). IL, illiterate

At baseline, significant sex differences were observed in ECG parameters: females had longer QTc intervals (430.3 ± 23.3 vs. 416.6 ± 23.9 ms, P < 0.001; mean difference +13.7 ms, 95% CI 9.9-17.6), whereas males had wider QRS duration (91.6 ± 10.2 vs. 86.0 ± 9.2 ms, P < 0.001) and longer PR intervals (150.1 ± 20.5 vs. 144.8 ± 18.5 ms, P = 0.001). Heart rate did not differ significantly (83.9 ± 17.0 vs. 85.8 ± 16.4 bpm, P = 0.176). The sex difference in baseline QTc was consistent across all five correction formulae (Bazett, Fridericia, Framingham, Hodges and Rautaharju; all P < 0.001; **Table 2**; **Figure 1**). In terms of metabolic parameters, females had significantly higher baseline CHOL (4.8 ± 1.0 vs. 4.3 ± 1.1 mmol/L, P < 0.001), HDL (1.5 ± 0.5 vs. 1.2 ± 0.3 mmol/L, P < 0.001), LDL (3.0 ± 0.8 vs. 2.8 ± 0.9 mmol/L, P = 0.041) and FBG (5.8 ± 1.4 vs. 5.6 ± 1.4 mmol/L, P = 0.030), while TG did not differ significantly (P = 0.100).

**Table 2:** Sensitivity analysis: QTc by sex across five heart-rate-correction formulae.

Correction formula	Baseline QTc, male	Baseline QTc, female	Baseline P	ΔQTc, male	ΔQTc, female	Crude Δ P	Adjusted female β (ms)	Adjusted P
Bazett	416.6 ± 23.9	430.3 ± 23.3	<0.001	−4.6 ± 22.9	−2.2 ± 25.4	0.219	+9.75	<0.001
Fridericia	395.1 ± 19.0	406.7 ± 22.6	<0.001	−0.8 ± 18.9	−0.1 ± 24.9	0.714	+7.14	<0.001
Framingham	395.8 ± 17.5	406.6 ± 20.9	<0.001	−0.2 ± 17.5	+0.3 ± 22.9	0.743	+6.48	<0.001
Hodges	398.4 ± 18.1	409.5 ± 18.7	<0.001	−2.8 ± 17.4	−1.4 ± 21.2	0.388	+7.28	<0.001
Rautaharju	401.6 ± 19.9	413.9 ± 21.4	<0.001	−2.7 ± 19.4	−1.3 ± 23.9	0.429	+8.10	<0.001

Adjusted female β = coefficient for female sex from ANCOVA of ΔQTc on sex, age, length of stay and baseline QTc (without drug-group terms; the fully adjusted multivariable model including drug group is given in **Table 3**). Because digital raw waveforms were unavailable, uncorrected QT was back-calculated from the automatically reported Bazett QTc; consequently absolute values for the alternative formulae should be interpreted with caution.

**Table 3:** Multivariable linear regression: factors associated with ΔQTc (post-treatment − pre-treatment).

Variable	β	95% CI	P value
Female sex	+9.91	+6.53 to +13.30	<0.001**
Age	+0.24	+0.09 to +0.39	0.002**
Length of stay	+0.03	−0.00 to +0.07	0.078
Baseline QTc	−0.54	−0.61 to −0.48	<0.001**
Clozapine (vs. risperidone)	+5.14	−0.75 to +11.03	0.088
Olanzapine (vs. risperidone)	+2.21	−3.65 to +8.07	0.460
Combination therapy (vs. risperidone)	+2.99	−1.52 to +7.51	0.194
Other drugs (vs. risperidone)	−2.72	−8.61 to +3.16	0.365

β, unstandardized coefficient; risperidone as reference; R² = 0.325, model P < 0.001. * P < 0.05; ** P < 0.01.

**Figure 1:**
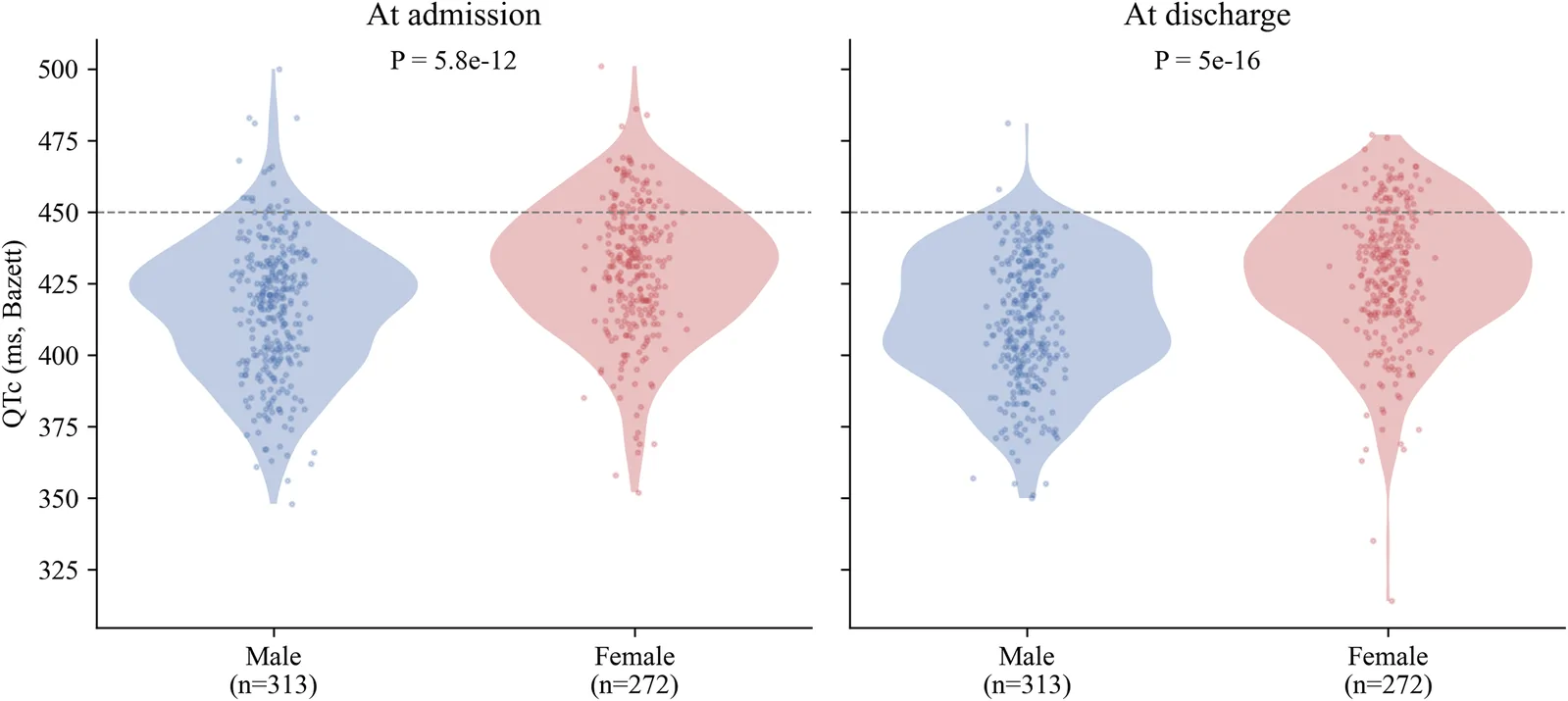
Distribution of QTc (Bazett) by sex at admission and at discharge. Violin plots with individual data points; the dashed line indicates 450 ms. (n indicates the number of patients with analysable ECGs).

### 3.2 Changes in ECG and metabolic parameters after antipsychotic treatment

**Table 4** presents the changes in ECG and metabolic parameters from admission to discharge, stratified by sex. Overall, heart rate decreased significantly in both sexes (males: −4.8 ± 15.9 bpm, P < 0.001; females: −2.7 ± 16.2 bpm, P = 0.007). QTc shortened significantly in males (−4.6 ± 22.9 ms, P = 0.0004) but not in females (−2.2 ± 25.4 ms, P = 0.165). PR interval and QRS duration showed no significant within-group changes in either sex.

**Table 4:** Changes in electrocardiographic and metabolic parameters before and after antipsychotic treatment by sex.

Variable	Male baseline	Male post	Male Δ	Male P	Female baseline	Female post	Female Δ	Female P	P (Δ M vs F)	P (ANCOVA)	P (sex × time, mixed)
Heart rate (bpm)	83.9 ± 17.0	79.1 ± 14.3	−4.8 ± 15.9	<0.001	85.8 ± 16.4	83.1 ± 14.8	−2.7 ± 16.2	0.007	0.108	0.008	0.107
QRS duration (ms)	91.6 ± 10.2	91.7 ± 9.9	+0.2 ± 6.6	0.611	86.0 ± 9.2	85.5 ± 8.7	−0.6 ± 7.6	0.227	0.204	<0.001	0.203
PR interval (ms)	150.1 ± 20.5	150.7 ± 20.1	+0.6 ± 12.8	0.390	144.8 ± 18.5	146.2 ± 20.1	+1.5 ± 14.4	0.093	0.450	0.565	0.451
QTc interval (ms)	416.6 ± 23.9	411.9 ± 22.6	−4.6 ± 22.9	0.0004	430.3 ± 23.3	428.1 ± 24.0	−2.2 ± 25.4	0.165	0.216	<0.001	0.215
Triglycerides (mmol/L)	1.6 ± 1.3	1.8 ± 1.2	+0.1 ± 1.2	0.067	1.5 ± 1.3	1.6 ± 1.1	+0.2 ± 1.3	0.009	0.540	0.455	0.466
Total cholesterol (mmol/L)	4.3 ± 1.1	4.3 ± 0.9	−0.1 ± 0.9	0.135	4.8 ± 1.0	4.8 ± 1.0	+0.0 ± 0.9	0.518	0.136	<0.001	0.135
HDL cholesterol (mmol/L)	1.2 ± 0.3	1.2 ± 0.3	−0.1 ± 0.3	<0.001	1.5 ± 0.5	1.4 ± 0.3	−0.1 ± 0.4	<0.001	0.012	0.003	0.012
LDL cholesterol (mmol/L)	2.8 ± 0.9	2.9 ± 1.3	+0.0 ± 1.3	0.549	3.0 ± 0.8	3.1 ± 0.8	+0.1 ± 0.7	0.067	0.661	0.275	0.661
Fasting blood glucose (mmol/L)	5.6 ± 1.4	5.1 ± 0.8	−0.5 ± 1.2	<0.001	5.8 ± 1.4	5.2 ± 1.0	−0.6 ± 1.1	<0.001	0.309	0.691	0.308

Paired t-test; independent t-test comparing Δ between sexes; ANCOVA adjusted for age, length of hospital stay and baseline value; mixed-effects model with participant as a random intercept (sex × time interaction).

In unadjusted analyses, the magnitude of change differed significantly between sexes only for HDL (males: −0.08 ± 0.27 vs. females: −0.14 ± 0.37 mmol/L, P = 0.012). However, after adjustment for age, length of stay and baseline values, ANCOVA revealed significant sex differences in the changes of QTc (P < 0.001), heart rate (P = 0.008), QRS duration (P < 0.001), HDL (P = 0.003) and CHOL (P < 0.001), indicating that sex was associated with differential treatment-related changes in these parameters after adjustment for baseline values. In mixed-effects models, the sex × time interaction did not reach significance for any ECG parameter (QTc, P = 0.215; heart rate, P = 0.107; QRS, P = 0.203; PR, P = 0.451); among the metabolic parameters only HDL showed a significant sex × time interaction (P = 0.012), which did not survive FDR correction (q = 0.108).

Both sexes showed significant decreases in HDL (P < 0.001 for both) and FBG (P < 0.001 for both). TG increased significantly in females (+0.19 ± 1.27 mmol/L, P = 0.009) and showed a trend in males (+0.13 ± 1.23 mmol/L, P = 0.067).

### 3.3 QTc prolongation prevalence and changes after treatment

**Table 5** shows the prevalence of QTc prolongation at admission and at discharge according to sex-specific thresholds together with 95% CIs and Fisher’s exact tests. At admission, the prevalence of marked QTc prolongation (male >450 ms, female >470 ms) was significantly higher in males than in females (5.4% [95% CI 3.4-8.5] vs. 1.5% [95% CI 0.6-3.7], Fisher exact P = 0.013; OR = 3.85, 95% CI 1.28-11.58). Similarly, QTc prolongation tendency (male >440 ms, female >460 ms) was more common in males at admission (15.0% [95% CI 11.5-19.4] vs. 8.8% [95% CI 6.0-12.8], Fisher exact P = 0.023; **Figure 2**).

**Figure 2:**
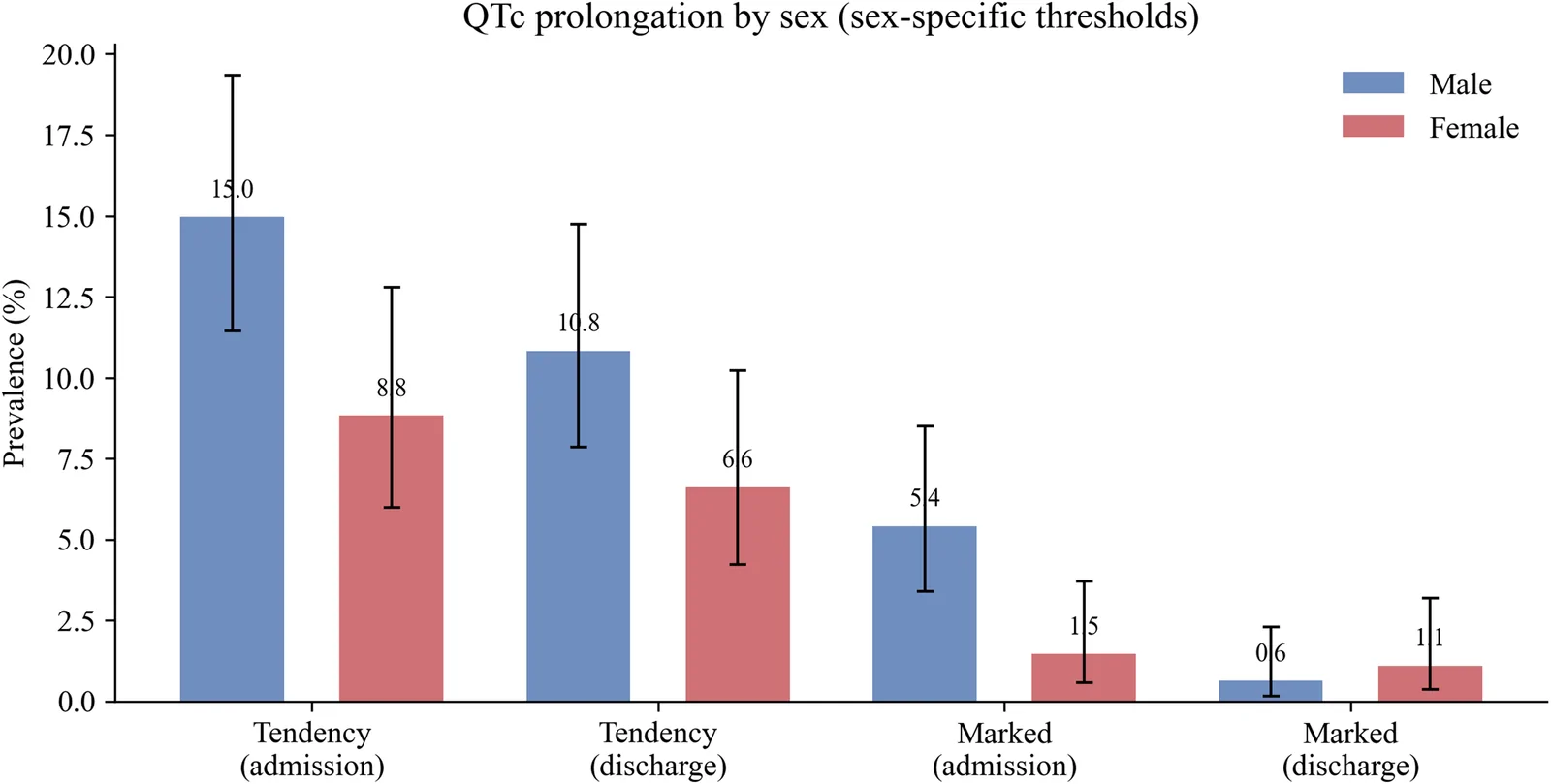
Prevalence of QTc prolongation by sex and time point, using sex-specific thresholds (prolongation tendency: male >440 ms, female >460 ms; marked prolongation: male >450 ms, female >470 ms). Error bars denote 95% confidence intervals (Wilson).

After antipsychotic treatment, the prevalence of marked QTc prolongation in males declined markedly from 5.4% to 0.6% (exact McNemar P = 0.0007), whereas in females it remained essentially unchanged (1.5% to 1.1%, exact McNemar P = 1.000). Consequently, the sex difference in marked QTc prolongation disappeared at discharge (0.6% vs. 1.1%, Fisher exact P = 0.668).

### 3.4 Regression analyses

**Table 6** shows the multivariable logistic regression for QTc prolongation tendency at admission (male >440/female >460 ms). Female sex was independently associated with lower odds of QTc prolongation tendency (OR = 0.49, 95% CI 0.29-0.85, P = 0.011) after adjusting for age, length of stay and antipsychotic drug group. Age, length of stay and drug group were not significantly associated with QTc prolongation tendency. For marked QTc prolongation, the low number of events (17 males, 4 females) precluded multivariable modeling; univariable analysis confirmed the higher risk in males (OR = 3.85, 95% CI 1.28-11.58, P = 0.019), and this estimate is also reported in **Table 5** rather than in a separate table.

**Table 5:** Prevalence of QTc prolongation and changes after treatment (with 95% CIs and Fisher's exact tests).

QTc prolongation definition	Time point	Male, n (%) [95% CI]	Female, n (%) [95% CI]	Fisher exact P
Prolongation tendency (M >440 / F >460 ms)	Pre-treatment (admission)	47 (15.0%) [11.5–19.4]	24 (8.8%) [6.0–12.8]	0.023
Prolongation tendency (M >440 / F >460 ms)	Post-treatment (discharge)	34 (10.8%) [7.9–14.8]	18 (6.6%) [4.2–10.2]	0.081
Marked prolongation (M >450 / F >470 ms)	Pre-treatment (admission)	17 (5.4%) [3.4–8.5]	4 (1.5%) [0.6–3.7]	0.013
Marked prolongation (M >450 / F >470 ms)	Post-treatment (discharge)	2 (0.6%) [0.2–2.3]	3 (1.1%) [0.4–3.2]	0.668
Male >450 ms (exact McNemar)	Pre → Post	17 (5.4%) → 2 (0.6%)	—	0.0007
Female >470 ms (exact McNemar)	Pre → Post	—	4 (1.5%) → 3 (1.1%)	1.000

Sex-specific thresholds were used (male >440/450 ms; female >460/470 ms). 95% CIs (Wilson) and Fisher's exact tests were added; exact McNemar tests were used for within-sex changes. The univariable sex difference in marked prolongation at admission is OR = 3.85 (95% CI 1.28–11.58).

**Table 6:** Multivariable logistic regression: risk factors for QTc prolongation tendency at admission (male >440 / female >460 ms).

Variable	OR	95% CI	P value
Female (vs. male)	0.49	0.29–0.85	0.011*
Age (per 1-year increase)	1.02	0.99–1.04	0.199
Length of stay (per 1-day increase)	0.99	0.99–1.00	0.079
Clozapine (vs. risperidone)	0.71	0.26–1.90	0.493
Olanzapine (vs. risperidone)	0.69	0.27–1.81	0.453
Combination therapy (vs. risperidone)	0.98	0.49–1.93	0.944
Other drugs (vs. risperidone)	1.08	0.45–2.59	0.861

Risperidone served as the reference category. OR, odds ratio; CI, confidence interval. * P < 0.05.

**Table 3** shows the multivariable linear regression for ΔQTc. Female sex was associated with significantly smaller QTc shortening (β = +9.91 ms, 95% CI 6.53-13.30, P < 0.001), indicating greater QTc shortening in males after adjusting for covariates. Higher baseline QTc was associated with greater shortening (β = −0.54, P < 0.001), whereas older age was associated with less shortening (β = +0.24, P = 0.002). No antipsychotic drug group differed significantly from risperidone in ΔQTc. The model explained 32.5% of the variance in ΔQTc (R² = 0.325, model P < 0.001).

### 3.5 Subgroup analysis by antipsychotic agent

**Table 7** shows sex differences in QTc parameters within major antipsychotic groups. Baseline QTc was significantly longer in females than in males among patients receiving olanzapine (435.2 ± 23.6 vs. 414.4 ± 20.5 ms, P < 0.001), risperidone (429.3 ± 24.3 vs. 415.6 ± 28.0 ms, P = 0.008) and combination therapy (430.6 ± 22.0 vs. 418.3 ± 23.4 ms, P < 0.001), but not among those receiving clozapine (427.3 ± 21.5 vs. 420.6 ± 19.1 ms, P = 0.155). No significant sex differences in ΔQTc were observed within any drug subgroup (**Table 7**).

**Table 7:** Subgroup analysis by antipsychotic agent: sex differences in QTc.

Drug group	n (M/F)	Baseline QTc, male	Baseline QTc, female	P	ΔQTc, male	ΔQTc, female	P
Clozapine	32/49	420.6 ± 19.1	427.3 ± 21.5	0.155	−1.0 ± 19.9	+4.8 ± 23.5	0.255
Olanzapine	41/32	414.4 ± 20.5	435.2 ± 23.6	<0.001**	−3.7 ± 15.2	−6.0 ± 21.9	0.600
Risperidone	57/51	415.6 ± 28.0	429.3 ± 24.3	0.008**	−8.5 ± 24.2	−4.3 ± 29.7	0.410
Combination therapy	147/106	418.3 ± 23.4	430.6 ± 22.0	<0.001**	−3.6 ± 23.5	−1.6 ± 25.6	0.525
Higher-QTc-risk regimen (clozapine/olanzapine) vs. others	344 vs 242	—	—	—	ΔQTc difference: β = +3.43 ms (P = 0.048, Bazett); −1.48 ms (P = 0.35, Fridericia)		

ΔQTc, post-treatment QTc minus pre-treatment QTc (ms). The final row shows the re-classification of combination therapy by the presence of a higher-QTc-risk agent; the between-sex difference in ΔQTc was unchanged when this binary exposure replaced the drug group.

### 3.6 Sensitivity and robustness analyses

#### 3.6.1 Rate-correction formulae

When QTc was recomputed with four alternative formulae, the baseline sex difference persisted for every formula (female minus male: Bazett +13.7, Fridericia +11.6, Framingham +10.8, Hodges +11.1 and Rautaharju +12.3 ms; all P < 0.001; **Table 2**). In contrast, the crude within-sex magnitude of QTc change was formula-dependent: males showed a statistically significant shortening under Bazett (−4.6 ms, P = 0.0004), Hodges (−2.8 ms, P = 0.005) and Rautaharju (−2.7 ms, P = 0.014), but not under Fridericia (−0.8 ms, P = 0.48) or Framingham (−0.2 ms, P = 0.83); females showed no significant shortening under any formula. The crude between-sex difference in ΔQTc was not statistically significant for any formula (all P > 0.2), yet the baseline-adjusted between-sex difference remained significant for every formula after adjustment for age, length of stay and baseline QTc (female β = +9.75, +7.14, +6.48, +7.28 and +8.10 ms for Bazett, Fridericia, Framingham, Hodges and Rautaharju, respectively; all P < 0.001; **Table 2**). Threshold-based prevalence was likewise formula-dependent: applying the same sex-specific cut-offs to Fridericia-corrected values reduced marked prolongation at admission to 0.6% in males and 0.0% in females (Fisher exact P = 0.50), indicating that the absolute prevalence of threshold-defined prolongation depends strongly on the correction formula (**Figure 3**).

**Figure 3:**
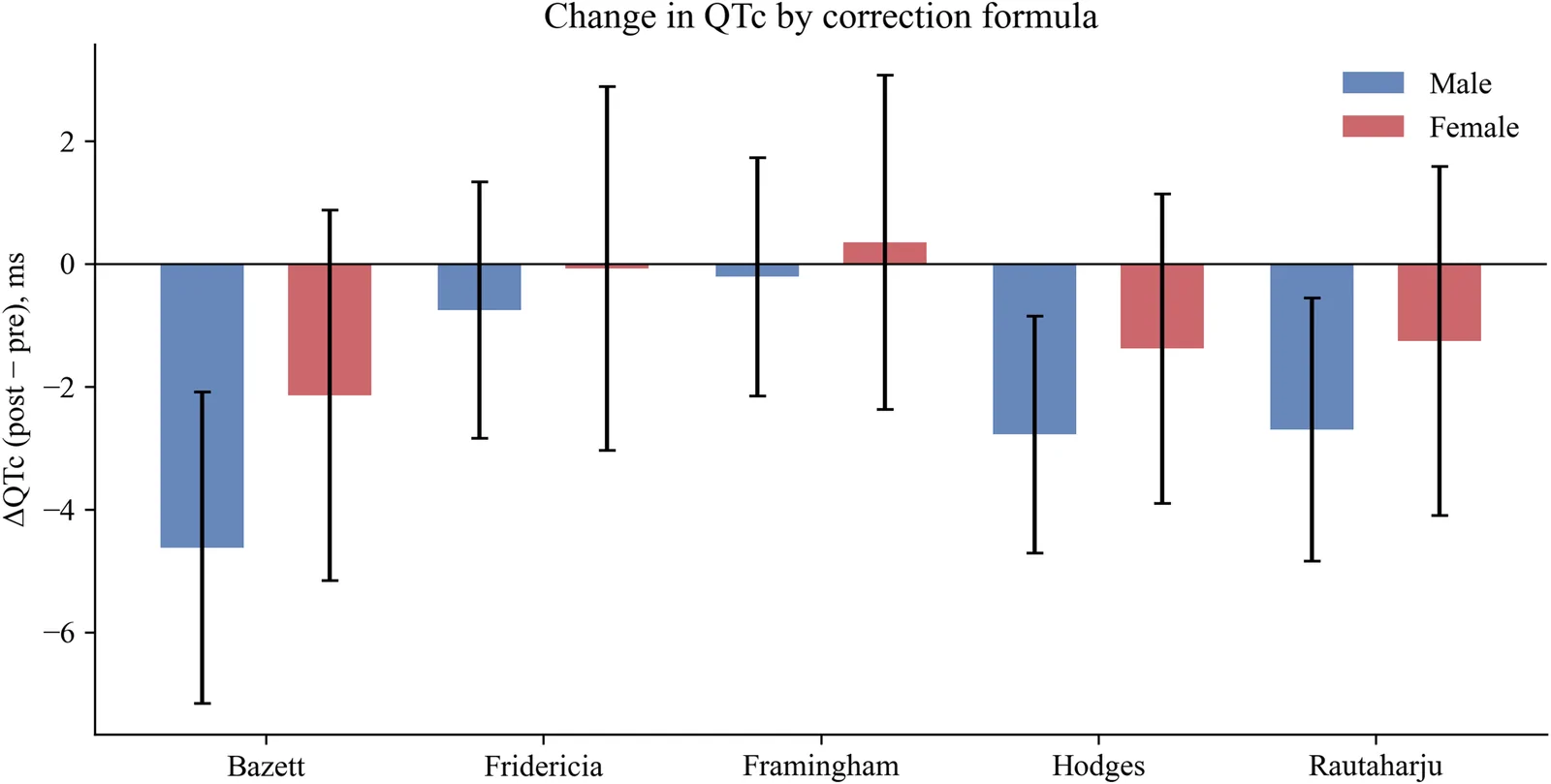
Mean change in QTc (post-treatment minus pre-treatment, ΔQTc) by sex across five heart-rate-correction formulae. Error bars denote 95% confidence intervals.

#### 3.6.2 Combination-therapy re-classification

The most common combination regimens were risperidone + clozapine (n = 58), risperidone + olanzapine (n = 49) and risperidone + aripiprazole (n = 28). Of the 253 patients receiving combination therapy, 192 (75.9%) received a regimen containing clozapine or olanzapine; overall, 344 of 586 patients (58.7%) received a higher-QTc-risk regimen. When the models were repeated using this binary exposure instead of the drug group, the between-sex difference in ΔQTc was essentially unchanged (Bazett: female β = +9.76 ms, P < 0.001; Fridericia: female β = +7.18 ms, P < 0.001), whereas the higher-QTc-risk class itself was only weakly associated with ΔQTc (Bazett β = +3.43 ms, P = 0.048; Fridericia β = −1.48 ms, P = 0.35).

#### 3.6.3 Multiple comparisons

Applying the Benjamini-Hochberg FDR procedure to the 11 baseline between-sex comparisons, all differences except heart rate and TG remained significant after correction (age, length of stay, QRS, PR, QTc, CHOL, HDL, LDL and FBG; all q < 0.05); among the 9 between-sex comparisons of change, none survived FDR correction (all q > 0.10), consistent with the unadjusted t-tests.

#### 3.6.4 Internal site proxy

Because the pooled dataset did not contain an explicit study-site code, medical-record numbers were used to derive an internal proxy of the two contributing hospitals; this proxy identified 62 patients (10.6%) with 6-digit record numbers and 524 patients (89.4%) with 4-5-digit record numbers. The direction of the baseline sex difference was consistent in both groups (female minus male: +6.9 ms in the smaller group and +14.1 ms in the larger group). This proxy is not a validated site variable and these exploratory results should be interpreted with caution.

## 4 Discussion

In this study of 586 hospitalized patients with SCZ, we characterized sex differences in ECG parameters and their changes during antipsychotic treatment. Three principal findings emerged. First, consistent with population physiology, females had significantly longer baseline QTc intervals, while males had wider QRS and longer PR intervals. Second, and more strikingly, when guideline-recommended sex-specific QTc thresholds were applied, the prevalence of marked QTc prolongation at admission was significantly higher in males, and antipsychotic treatment was associated with a pronounced reduction in this proportion among males but no significant change in females. Third, after adjustment for baseline and clinical covariates, sex independently modulated QTc changes during treatment, with males showing significantly greater QTc shortening.

Our finding that females had longer baseline QTc intervals than males aligns with well-established population studies (11, 26). This difference arises primarily from androgen-associated shortening of ventricular repolarization in postpubertal males rather than prolongation in females (27, 28). Consistent with recent ECG reference studies, males also had wider QRS duration and longer PR intervals in our cohort (29, 30), reflecting sex differences in cardiac mass, conduction system physiology and autonomic tone. These baseline differences underscore the importance of sex-specific interpretation of ECG parameters in clinical practice and research (31, 32).

A notable finding of our study is that, despite longer mean QTc in females, the prevalence of marked QTc prolongation defined by sex-specific thresholds was higher in males at admission. This apparent paradox is explained by the relationship between the sex difference in mean QTc and the sex difference in thresholds. Because the threshold gap exceeds the mean difference, proportionally more males exceed their sex-specific threshold. Previous studies in SCZ populations—mostly cross-sectional and conducted in chronically institutionalized or community patients—have generally reported a higher prevalence of QTc prolongation in females (15, 16). The discrepancy with our findings likely reflects several factors: differences in disease phase, treatment settings, threshold definitions, and the age distribution. Our cohort was restricted to patients aged 18–60 years, an age range in which the sex difference in QTc is most pronounced (9, 33, 34); menopausal status was not recorded, so some women may have been perimenopausal or postmenopausal. Our results suggest that in acutely hospitalized Chinese patients with SCZ, male patients may carry a higher burden of threshold-defined QTc prolongation at admission and warrant particular ECG attention at treatment initiation.

Several mechanisms may contribute to the observed between-sex difference in QTc trajectory. Importantly, our sensitivity analyses showed that the crude magnitude of within-male QTc shortening was strongly dependent on the correction formula: it was significant under formulae that retain residual heart-rate dependence (Bazett, and to a lesser extent Hodges and Rautaharju) but not under Fridericia or Framingham, a pattern consistent with part of the apparent male shortening being an artefact of males’ larger reduction in heart rate (24, 25). The crude between-sex difference in ΔQTc was not statistically significant for any formula; the sex difference emerged only after adjustment for baseline QTc, which differed between the sexes, and remained robust across all five formulae. Baseline QTc strongly predicted ΔQTc, consistent with regression to the mean and with repolarization normalization among patients with elevated baseline QTc, who were predominantly male in our cohort. Other contributors may include the improvement of clinical status with reduced agitation-related sympathetic activation and lower heart rate, and sex differences in antipsychotic pharmacokinetics: women often achieve higher dose-corrected blood concentrations of several antipsychotics, which may translate into differential QTc effects even at similar prescribed doses (19, 20, 35). Sex hormones modulate cardiac repolarization dynamically: androgens shorten, whereas estrogens prolong, ventricular repolarization, and the hormonal milieu may influence drug-ion channel interactions (9, 10). Interestingly, a previous study found that switching from olanzapine to risperidone shortened QTc significantly only in females (36), highlighting that drug-sex interactions in QTc response are complex and setting-dependent. Taken together, these observations indicate that the between-sex difference in QTc change in our cohort should be interpreted cautiously and confirmed prospectively. Adjustment for age and length of stay did not materially change these estimates (for Bazett QTc, the baseline-adjusted female β was +9.14 ms without and +9.75 ms with length of stay), and the sex difference in length of stay (males 90.2 vs. females 78.0 days) was not explained by clozapine titration: length of stay was similar in both sexes among clozapine-treated patients (100.4 vs. 102.4 days, P = 0.78) and differed only in those not receiving clozapine (females 64.7 vs. males 85.1 days), more probably reflecting differences in clinical presentation and discharge planning.

Our findings have practical implications for cardiovascular safety monitoring in SCZ. Current guidelines recommend baseline and follow-up ECG for patients starting or switching antipsychotics, with particular attention to QTc >500 ms or increases ≥60 ms (4–6). Sex-specific thresholds (males >450 ms, females >470 ms) are recommended for interpreting QTc in drug safety contexts (4). Our results suggest that male inpatients with SCZ, despite longer baseline QRS and PR intervals and lower mean QTc, may warrant particular ECG attention at admission, as they showed the highest prevalence of threshold-defined marked QTc prolongation. Conversely, female patients had persistently longer QTc intervals and, in adjusted models, less QTc shortening during treatment, which may warrant continued monitoring beyond the acute phase. These considerations are particularly relevant given that sudden cardiac death in SCZ shows notable age- and sex-specific patterns (37, 38). However, given the observational design and the absence of clinical outcome data, our findings should be regarded as hypothesis-generating and require prospective confirmation.

Females in our cohort had higher baseline CHOL, HDL, LDL and FBG, consistent with previous reports of a greater obesity and dyslipidemia burden in female patients with SCZ; sex differences in antipsychotic-induced metabolic changes, however, vary by drug and parameter, with some studies reporting greater glucose and lipid alterations in men receiving clozapine or olanzapine (39, 40). Sex differences in antipsychotic-induced metabolic disturbances are well documented; however, the direction and magnitude of these differences may depend on the drug, dose and metabolic parameter examined (19, 35). Our metabolic assessment was limited to a single fasting blood glucose value and a standard lipid panel at two time points, without glycated hemoglobin (HbA1c), insulin or homeostatic model assessment of insulin resistance (HOMA-IR); therefore, the term “metabolic assessment” should be regarded as a limited screening of glucose and lipid status rather than a comprehensive metabolic evaluation, and acute illness or stress could transiently affect these values. The higher baseline lipid levels in females may partly reflect the older age of female patients in our cohort, but the sex-specific HDL trajectory after adjustment suggests an independent treatment-related effect. These metabolic sex differences, together with the ECG findings, reinforce calls for sex-stratified cardiometabolic monitoring during antipsychotic treatment (19, 35, 40).

In subgroup analyses, baseline QTc sex differences were significant in olanzapine, risperidone and combination therapy groups but not in the clozapine group. Clozapine is associated with substantial cardiovascular risks, including myocarditis and cardiomyopathy (41), and its QTc-prolonging effect has been linked to higher blood concentrations (42); such risks may attenuate the baseline sex differences expected in this subgroup. The relatively small sample size of the clozapine subgroup limits the interpretation. In the multivariable analysis, none of the antipsychotic drug groups differed significantly from risperidone in ΔQTc, although clozapine showed a trend toward less QTc shortening. Because combination therapy accounted for nearly half of the cohort and individual antipsychotics differ in QTc liability, we repeated the analyses after re-classifying regimens by the presence of a higher-QTc-risk agent (clozapine or olanzapine); the between-sex difference in ΔQTc was unchanged, whereas the higher-risk class itself was only weakly associated with ΔQTc. Thus, the null between-drug results are unlikely to be explained solely by the pooling of combination regimens; confounding by indication and dosing cannot be fully excluded in an observational analysis.

## 5 Limitations

This study has several limitations. First, as a retrospective, single-dataset observational study, residual confounding by unmeasured factors cannot be entirely excluded, and analyses were adjusted only for key demographic and clinical covariates; the two groups also differed at baseline in age (41.6 vs. 44.6 years) and length of stay (90.2 vs. 78.0 days), and although both were included as covariates, their influence on the conclusions cannot be fully excluded. Second, several variables potentially relevant to QTc—body mass index, serum electrolytes, antipsychotic blood concentrations, menopausal status, smoking, alcohol use, physical activity and concomitant use of other autonomically active drugs such as benzodiazepines—were incomplete or not collected, and study site was not recorded as an explicit variable, so site-level differences in acquisition or coding cannot be formally excluded, although the direction of the baseline sex difference was consistent across an internally derived site proxy. Third, most patients were already receiving antipsychotics at admission and treatment was adjusted naturalistically, so the admission ECG does not represent a medication-naive baseline, and QTc changes may partly reflect drug switching, dose titration or adjunctive treatment rather than treatment initiation. Fourth, QT/QTc was measured by the automated algorithm of the electrocardiograph with clinician over-reading rather than by a single blinded core laboratory or a defined manual T-wave-end rule, and different delineation algorithms can yield systematically different QT values (21, 22, 25); because raw digital waveforms and beat-to-beat recordings were unavailable, we could not compare measurement methods or assess the QT–RR relationship dynamically (i.e., QT/RR hysteresis), which depends on the history of preceding RR intervals and on factors independent of past RR intervals (43–46). Fifth, because Bazett’s formula retains residual heart-rate dependence, the greater heart-rate reduction in males may have contributed to their larger apparent QTc shortening, although the baseline-adjusted between-sex difference persisted across all formulae; the metabolic evaluation was also limited to fasting glucose and a lipid panel, without HbA1c, insulin or HOMA-IR. Finally, multiple comparisons were performed—although FDR correction was applied and the analyses are framed as exploratory, residual false-positive findings remain possible—clinical arrhythmic outcomes were not available, and the predominantly middle-aged inpatient population may limit generalizability.

## 6 Conclusion

In hospitalized patients with SCZ, we observed significant sex differences in ECG parameters and their trajectories during antipsychotic treatment. Although female patients had longer baseline QTc intervals across all correction formulae, male patients had a higher prevalence of marked QTc prolongation defined by sex-specific thresholds at admission and showed greater QTc shortening in baseline-adjusted models; however, the crude magnitude of QTc change was formula-dependent and the between-sex difference was not significant in unadjusted analyses, so this finding should be considered exploratory. Female patients had persistently longer QTc intervals and no significant QTc shortening during treatment. These findings highlight the importance of sex-stratified ECG monitoring during acute antipsychotic treatment and call for prospective studies integrating validated study-site information, sex-specific thresholds, detailed drug-exposure data, dynamic QT–RR assessment and clinical outcomes.

## Data Availability

The original contributions presented in the study are included in the article/supplementary material. Further inquiries can be directed to the corresponding authors.
